# Targeting STAT3 signaling using stabilised sulforaphane (SFX-01) inhibits endocrine resistant stem-like cells in ER-positive breast cancer

**DOI:** 10.1038/s41388-020-1335-z

**Published:** 2020-05-30

**Authors:** Bruno M. Simões, Angélica Santiago-Gómez, Chiara Chiodo, Tiago Moreira, Daniel Conole, Scott Lovell, Denis Alferez, Rachel Eyre, Katherine Spence, Aida Sarmiento-Castro, Bertram Kohler, Ludivine Morisset, Marilena Lanzino, Sebastiano Andò, Elisabetta Marangoni, Andrew H. Sims, Edward W. Tate, Sacha J. Howell, Robert B. Clarke

**Affiliations:** 1 Breast Biology Group, Manchester Breast Centre, Division of Cancer Sciences, Faculty of Biology, Medicine and Health, University of Manchester, Manchester, UK; 2 Department of Pharmacy, University of Calabria, Arcavacata di Rende, Italy; 3 Molecular Sciences Research Hub, Imperial College, London, UK; 4 Institut Curie, PSL Research University, Translational Research Department, Paris, France; 5 Applied Bioinformatics of Cancer Group, University of Edinburgh Cancer Research UK Centre, Edinburgh, UK; 6 Department of Medical Oncology, The Christie NHS Foundation Trust, Manchester, UK

**Keywords:** Breast cancer, Hormone receptors, Cancer stem cells

## Abstract

Estrogen receptor (ER) positive breast cancer is frequently sensitive to endocrine therapy. Multiple mechanisms of endocrine therapy resistance have been identified, including cancer stem-like cell (CSC) activity. Here we investigate SFX-01, a stabilised formulation of sulforaphane (SFN), for its effects on breast CSC activity in ER+ preclinical models. SFX‐01 reduced mammosphere formation efficiency (MFE) of ER+ primary and metastatic patient samples. Both tamoxifen and fulvestrant increased MFE and aldehyde dehydrogenase (ALDH) activity of patient-derived xenograft (PDX) tumors, which was reversed by combination with SFX‐01. SFX-01 significantly reduced tumor-initiating cell frequency in secondary transplants and reduced the formation of spontaneous lung micrometastases by PDX tumors in mice. Mechanistically, we establish that both tamoxifen and fulvestrant induce STAT3 phosphorylation. SFX-01 suppressed phospho‐STAT3 and SFN directly bound STAT3 in patient and PDX samples. Analysis of ALDH+ cells from endocrine-resistant patient samples revealed activation of STAT3 target genes *MUC1* and *OSMR*, which were inhibited by SFX-01 in patient samples. Increased expression of these genes after 3 months’ endocrine treatment of ER+ patients (*n* = 68) predicted poor prognosis. Our data establish the importance of STAT3 signaling in CSC-mediated resistance to endocrine therapy and the potential of SFX-01 for improving clinical outcomes in ER+ breast cancer.

## Introduction

Three out of four cases of breast cancer (BC) express the estrogen receptor alpha (ER) and are treated with endocrine therapies, such as selective ER modulators (e.g., tamoxifen), aromatase inhibitors (e.g., letrozole), and selective ER down-regulators (e.g., fulvestrant) [1]. However, despite the undoubted success of endocrine treatments, distant BC recurrences and death occur at a steady rate for at least 15 years after the 5–10 year treatment period is completed [2]. This finding stresses the need for new approaches that can provide long-term disease-free survival.

Endocrine resistance hampers the cure of ER+ BC, and therefore major efforts have been employed to address the mechanisms. Acquired resistance can be mediated by modulation of ER activity through its mutation, by up-regulation of ER coactivators (e.g., FOXA1), by activation of mitogenic signaling pathways (e.g. MAPK, PI3K/AKT) induced by receptor tyrosine kinase activity (e.g., EGFR, HER2) or by overexpression of substrates for cyclin-dependent kinases (CDKs) [3, 4]. Several drugs targeting these pathways have been tested in clinical studies, and recently CDK4/6 kinase inhibitors have been shown to increase overall survival (OS) of patients with advanced ER+ BC and three (palbociclib, ribociclib and abemaciclib) are now FDA approved [5–7].

Cancer stem cells (CSCs) can be responsible for tumor initiation and growth and are more resistant than non-CSCs to cancer therapies, such as chemo- and radiotherapy [8]. We and others have shown that breast CSCs (measured by the percentage of ALDH+, or mammosphere-forming cells) are not targeted by endocrine therapies in ER+ BC [9–11]. This leads to enrichment in cells with breast CSC activity which are dependent upon developmental signaling pathways, such as Notch and Wnt [12, 13]. Eradication of endocrine-resistant CSCs is likely to provide long-term disease-free survival but so far none of the approved drugs for patients with ER+ tumors has been shown to target CSCs.

Sulforaphane (SFN), an isothiocyanate found in cruciferous vegetables, has demonstrated activity against breast CSCs [14], although clinical application has been hampered due to its inherent physicochemical and biological instability [15]. In order to improve its stability, SFX‐01, SFN formulated within an alpha-cyclodextrin complex, has been developed [16].

Here, we report that SFX‐01 used in combination with endocrine therapies prevents breast CSC enrichment in patient samples and PDX tumors in vivo, and mechanistically it directly targets STAT3 to inhibit its activity in endocrine resistance. STAT3 activation in patient primary tumors measured by expression of STAT3-induced genes predicts resistance to endocrine treatments.

## Results

### SFX-01 reduces breast CSC activity in primary and metastatic ER+ breast cancer patient derived-samples

**Fig. 1:**
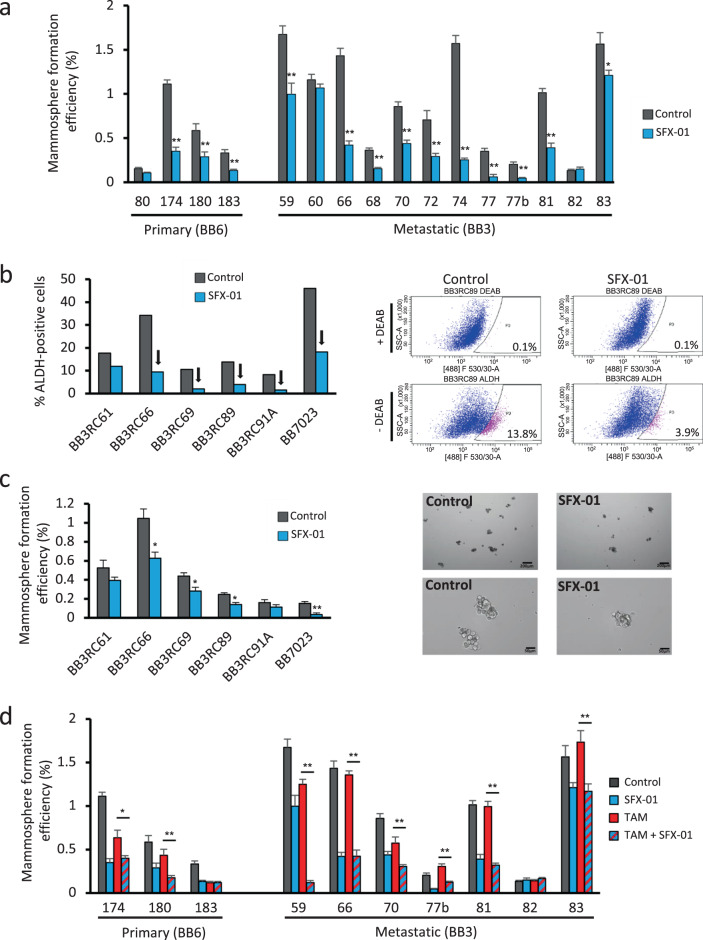
SFX-01 reduces breast CSC activity in primary and metastatic ER+ breast cancer patient derived-samples. **a** Mammosphere formation efficiency (MFE) of freshly isolated ER+ early (primary) and metastatic (pleural effusions and ascites) patient-derived samples cultured in the presence of SFX-01 (5 μM) or vehicle control. MFE data for each individual patient sample is represented. MFE was determined on days 7–9 and calculated by dividing the number of mammospheres formed (≥50 μm diameter) by the original number of single cells seeded (500 cells/cm^2^) and is expressed as the mean percentage of mammosphere formation. **b** Percentage of ALDH-positive cells in six ER+ metastatic BC patient-derived samples. Cells were grown in low-adherent plates in the presence of SFX-01 (5 μM) or water (control) for 72 h and then subjected to ALDEFLUOR assay. Arrows indicate a reduction greater than 60% compared with control. Representative FACS plots of ALDEFLUOR assay are shown. ALDH-positive cells were discriminated from ALDH-negative cells using the ALDH inhibitor, DEAB. **c** Percentage of MFE in six ER+ metastatic BC patient-derived samples. Cells were pre-treated in low-adherent plates in the presence of SFX-01 (5 μM) or water (control) for 72 h and then plated for MFE colony assay without drugs. Representative micrographs of mammospheres are shown. **d** Percentage of MFE of ER+ early (primary) and metastatic patient-derived samples treated with control (water and ethanol), SFX-01 (5 μM), tamoxifen (1 μM) or a combination of SFX-01 with tamoxifen. MFE data are represented as mean percentage ± SEM. *p < 0.05; **p < 0.01.

Mammosphere forming efficiency (MFE) and ALDH1 enzymatic activity have previously been shown to be a characteristic of breast CSCs [17, 18], which can be targeted by SFN in BC cell lines [14]. SFX-01 (SFN stabilised in alpha-cyclodextrin complex, Fig. S1a) reduced MFE in 13 of 16 patient-derived ER+ tumor samples (Fig. 1a and Supplementary Tables 1 and 2 for patient and tumor characteristics). Next, we assessed CSC activity following SFX-01 pre-treatment of cells from patients with endocrine-resistant BC. SFX-01 reduced the percentage of ALDH+ cells in all six samples (and by >60% in five; Fig. 1b) and also significantly decreased MFE in four of these samples (Fig. 1c). These data suggest that short term SFX-01 treatment targets breast CSC activity in patient-derived and endocrine-resistant cells.

We then investigated the effect of SFX-01 on MFE of patient-derived ER+ tumor cells cotreated with tamoxifen. SFX-01 remained effective and significantly reduced MFE in combination with tamoxifen in eight out of ten samples tested (Fig. 1d). In addition, we tested 3-day pre-treatment of ER+ cell lines (MCF-7, T47D, and ZR-75-1) with tamoxifen or fulvestrant in combination with SFX-01. Both ALDH activity and MFE were reduced by SFX-01 in all cell lines confirming reversal of the breast CSC activity that is induced by short-term treatment with antiestrogen drugs (Fig. S1b, c). Moreover, we have confirmed that the effects on MFE and ALDH positivity of commercially available SFN are comparable to the effects of SFX-01 at equivalent molar sulforaphane concentration (data not shown).

### SFX-01 prevents tamoxifen enrichment for cells with cancer stem cell properties in patient-derived xenograft tumors

**Fig. 2:**
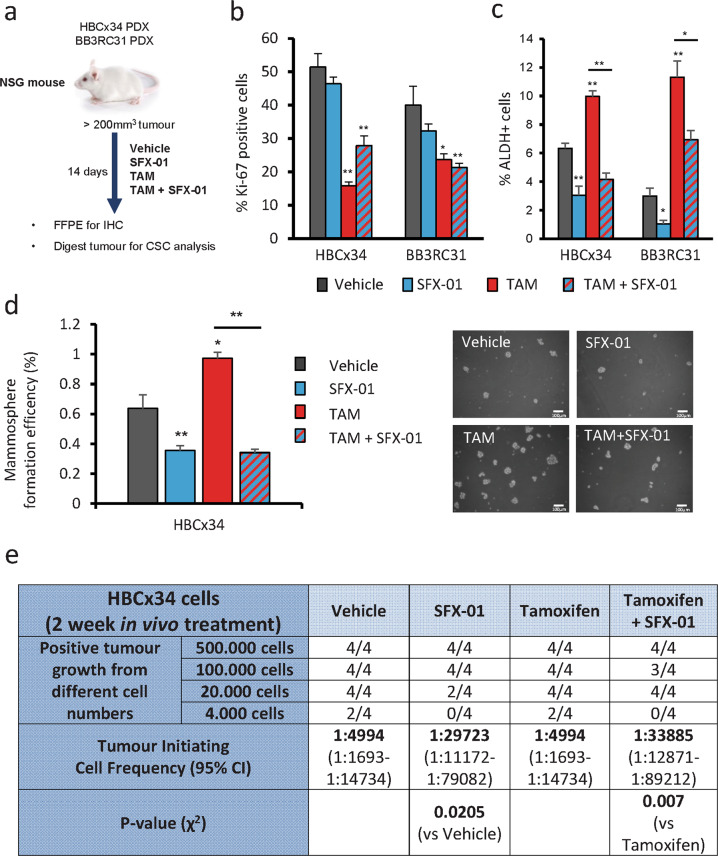
SFX-01 prevents tamoxifen enrichment for cells with cancer stem cell properties in patient-derived xenograft tumors. **a** BB3RC31 and HBCx34 patient derived xenografts (PDXs) treated in vivo for 14 days with SFX-01 (300 mg/kg/day, oral gavage) in the presence or absence of tamoxifen (10 mg/kg/day, oral gavage). HBCx34 model was kindly provided by Dr Elisabetta Marangoni (Institute Curie, Paris). Vehicle control used was 1% carboxymethylcellulose dissolved in distilled water. **b** Quantification of Ki67 expression determined by immunohistochemistry showing that tamoxifen but not SFX-01 significantly decreases proliferation marker Ki67. **c** Percentage of ALDH-positive cells was determined with ALDEFLUOR assay. ALDH-positive cells were discriminated from ALDH-negative cells using the ALDH inhibitor, DEAB. Mouse cells were excluded from the FACS analysis with anti-mouse MHC Class I (H-2K^d^) antibody. **d** Mammosphere formation efficiency was determined on days 7–9 and calculated by dividing the number of mammospheres formed (≥50 μm diameter) by the original number of single cells seeded (500 cells/cm^2^) and is expressed as the mean percentage of mammosphere formation. Representative micrographs are shown. **e** Secondary transplantation of 500, 100, 20, and 4 K cells after in vivo treatments. Experiment was carried out in NSG mice with 90-day slow-release estrogen pellets. Tumor growth (>75 mm^3^) was assessed at day 90 and is represented as mice positive for growth/mice tested for each cell number tested. ELDA of tumor-initiating cell frequency is shown. Data are represented as mean ± SEM. *p < 0.05; **p < 0.01.

To better model the clinical scenario of our treatments, two ER+ patient-derived xenograft (PDX) models were grown subcutaneously in NSG mice, one from an early (HBCx34, [19]) and another from a metastatic tumor (BB3RC31, [20]). We assayed proliferation and breast CSC activity in these PDX models after 14-day in vivo treatment with either SFX-01, tamoxifen, or combination of both drugs (Fig. 2a). We observed that tamoxifen treatment decreases the proliferation marker Ki67 and tumor growth in both PDX models and SFX-01 reduced tumor growth in the HBCx34 model, but had no significant impact on BB3RC31 (Figs. 2b and S2a). However, breast CSC activity measured by ALDH enzymatic activity or MFE was increased by tamoxifen and decreased by SFX-01, both alone and in combination with tamoxifen (Fig. 2c, d). We then performed in vivo limiting dilution transplantation of HBCx34 PDX cells derived from tumors previously treated with tamoxifen and/or SFX-01. Cells from tumors treated with SFX-01 could not form any tumors on reimplantation of 4,000 cells, in contrast with cells from tumors treated with vehicle control or tamoxifen alone (Fig. 2e). Graphs representing tumor size observed in all serial dilutions are shown in Fig. S2b. Overall, tumors treated with SFX-01 had reduced tumor-initiating cell frequency confirming that SFX-01 targets breast CSC activity in vivo (Fig. 2e).

### SFX-01 inhibits PDX tumor growth, prevents CSC activity and prevents spontaneous metastasis to the lungs

**Fig. 3:**
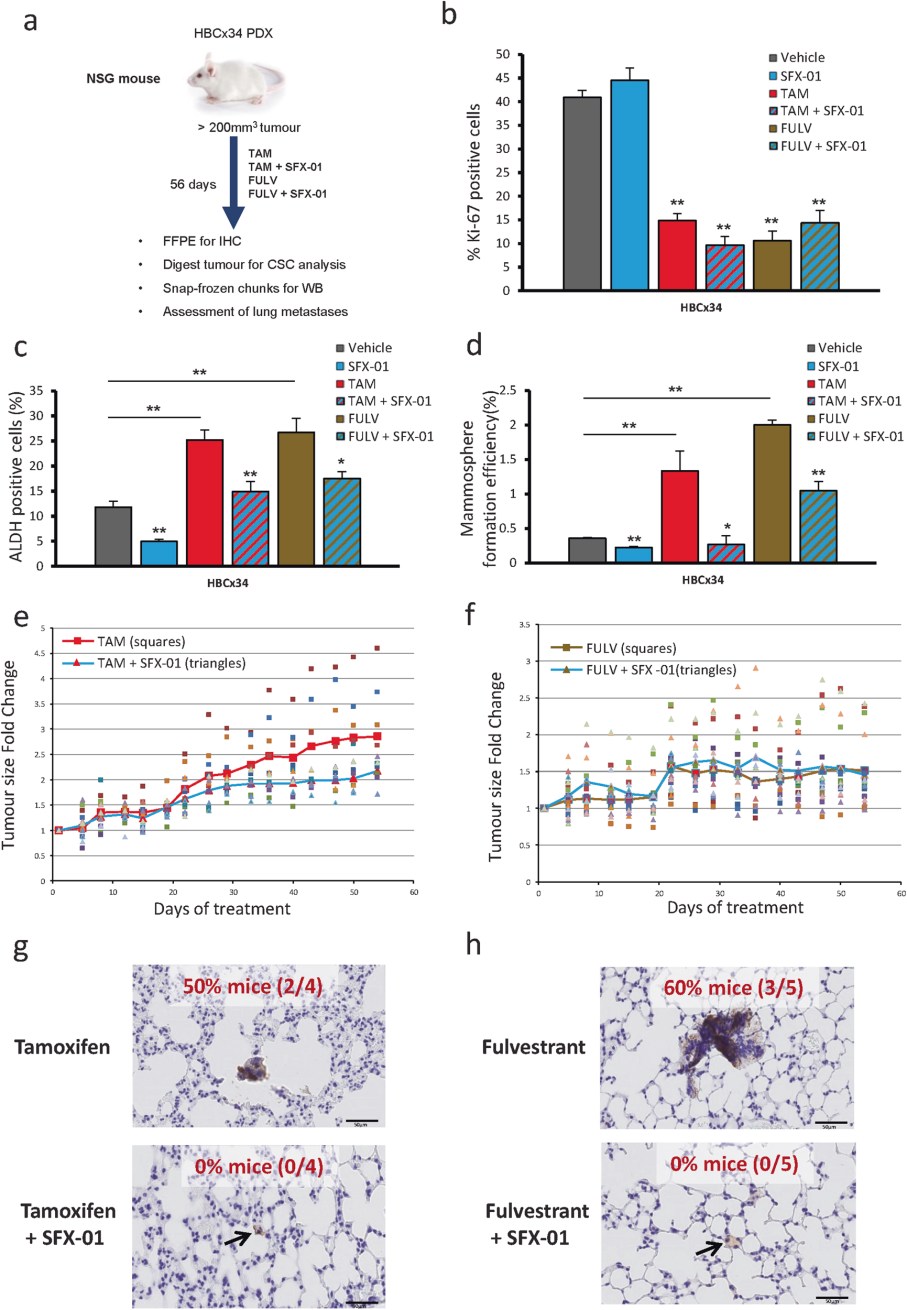
SFX-01 prevents CSC activity of PDX cells treated with tamoxifen and fulvestrant. SFX-01 inhibits tumor growth compared with tamoxifen alone and prevents formation of micrometastases in the lungs. **a** HBCx34 PDX treated in vivo for 56 days with tamoxifen (10 mg/kg/day, oral gavage) or fulvestrant (200 mg/kg/week, subcutaneous injection) in the presence or absence of SFX-01 (300 mg/kg/day, oral gavage). Vehicle control used was 1% carboxymethylcellulose in combination with a weekly subcutaneous injection of the fulvestrant vehicle. **b** Quantification of Ki67 expression determined by immunohistochemistry. **c** Percentage of ALDH-positive cells was determined with ALDEFLUOR assay. ALDH-positive cells were discriminated from ALDH-negative cells using the ALDH inhibitor, DEAB. Mouse cells were excluded from the FACS analysis with anti-mouse MHC Class I (H-2K^d^) antibody. **d** Mammosphere formation efficiency was determined on days 7–9 and calculated by dividing the number of mammospheres formed (≥ 50μm diameter) by the original number of single cells seeded (500 cells/cm^2^) and is expressed as the mean percentage of mammosphere formation. **e**–**f** Tumor growth of HBCx34 PDX tumors treated in vivo for 56 days. Individual tumors (*n* = 10) treated with tamoxifen (**e**) or fulvestrant (**f**) are represented by squares and tumors treated in combination with SFX-01 are represented by triangles. Red line shows average tumor growth for tamoxifen (**e**) or fulvestrant (**f**) and blue line shows average tumor growth for the combination treatment with SFX-01. **g**–**h** Mice lungs were stained with anti-human mitochondrial antibody and micrometastases with at least 10 cells were counted. Percentage of mice bearing micrometastases for each tamoxifen (**g**) and fulvestrant (**h**) treatment group is shown. Data are represented as mean ± SEM. *p < 0.05; **p < 0.01.

To further validate the in vivo effects of combining SFX-01 with antiestrogens on CSC activity we treated HBCx34 PDX cells for 56 days with either tamoxifen or fulvestrant (Fig. 3a). As expected, both antiestrogens strongly reduced the number of proliferative cells measured by Ki67 expression (Fig. 3b) whilst increasing both ALDH activity and MFE (Fig. 3c, d). SFX-01 had no effect on proliferation (Fig. 3b) but significantly inhibited antiestrogen stimulated breast CSC activity (Fig. 3c, d). A 56-day treatment of the second PDX model (BB3RC31) also demonstrated that the antiestrogen enrichment of breast CSCs can be reduced by SFX-01 (Fig. S3a, b). Over the 8-week treatment period, tamoxifen plus SFX-01 significantly suppressed tumor growth versus tamoxifen alone in the HBCx34 (Fig. 3e) but not the BB3RC31 PDX models (data not shown). On the other hand, fulvestrant treatment maintained tumor growth suppression at 8 weeks and no additive effect was seen with SFX-01 in either HBCx34 (Fig. 3f) or BB3RC31 (data not shown) PDX models.

We next investigated whether treatments had an effect on PDX spontaneous lung metastases. Using a human-specific mitochondrial antibody we could clearly observe micrometastases in the lungs from mice bearing HBCx34 PDX tumors treated with tamoxifen or fulvestrant (Fig. 3g, h). In contrast, lungs from HBCx34 mice treated with tamoxifen or fulvestrant in combination with SFX-01 were free from micrometastases, although disseminated tumor cells persisted (Fig. 3g, h). In the BB3RC31 PDX model SFX-01 reduced micrometastases by 50% in combination with fulvestrant but no effect was seen in combination with tamoxifen (Fig. S3c, d), suggesting SFX-01 hinders colonisation of the lungs by disseminated tumor cells in sensitive tumors.

### SFX-01 targets STAT3 signaling, which is activated by antiestrogen therapy

**Fig. 4:**
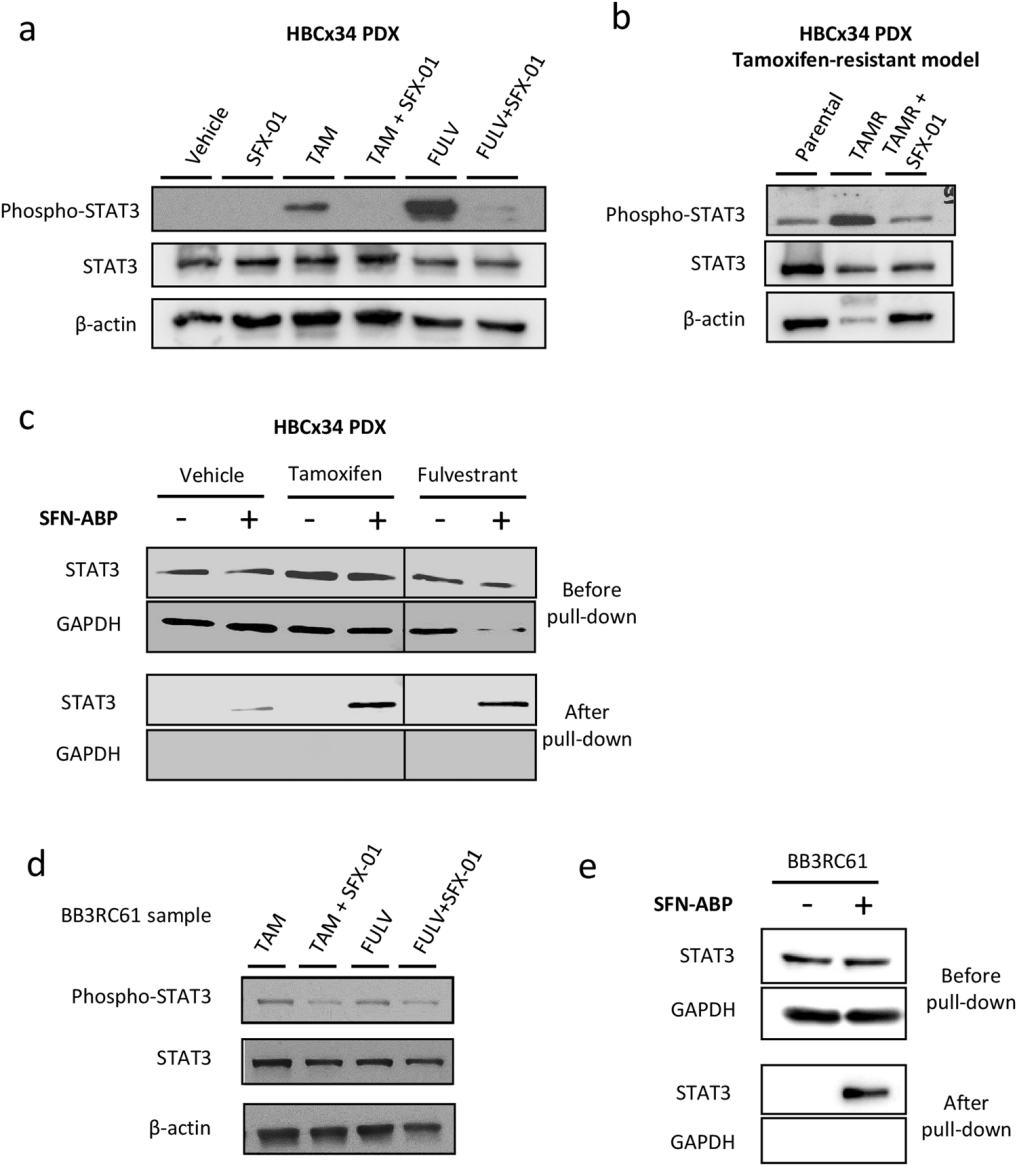
SFX-01 targets STAT3 signaling, which is activated by antiestrogen therapy. **a** phospho-STAT3 and total STAT3 protein expression levels determined by Western Blot in HBCx34 PDX treated in vivo for 56 days with tamoxifen or fulvestrant in the presence or absence of SFX-01. β-actin was used as a reference for the loading control. **b** Protein expression levels in tamoxifen-resistant (TAMR) HBCx34 PDX treated in vivo for 56 days with SFX-01. **c** Total STAT3 protein detection before and after SFN-ABP pull-down experiments in HBCx34 PDX treated tumors. GAPDH was used as a reference for the loading control. **d** Protein expression levels in metastatic patient-derived sample BB3RC61 treated for 72 h with tamoxifen or fulvestrant in the presence or absence of SFX-01. **e** Total STAT3 protein detection before and after SFN-ABP pull-down experiments in BB3RC61 sample. GAPDH was used as a reference for the loading control.

We next sought to determine the mechanism of sensitivity to SFX-01. The direct binding targets of SFN have been assessed in BC cell lines, identifying STAT3 and NF-κB subunits among the top high-affinity targets [21]. STAT3 was a particularly interesting target since ALDH+ breast CSCs and tamoxifen resistant cells have previously been shown to express higher levels of phospho-STAT3 (active form), and inhibition of STAT3 reduces ALDH+ cell numbers and tumorigenicity [22, 23]. HBCx34 PDX tumors treated in vivo for 56 days with tamoxifen or fulvestrant showed increased phospho-STAT3 expression which was inhibited by cotreatment with SFX-01 (Fig. 4a). In the BB3RC31 PDX model we also observed that fulvestrant, but not tamoxifen, increased phospho-STAT3 expression and SFX-01 only reduced phospho-STAT3 levels in fulvestrant-treated tumors (Fig. S4b). We also assessed whether phospho-NF-κB p65 was modulated in a similar way to STAT3 but overall its expression did not change in either HBCx34 or BB3RC31 PDX treated tumors (Fig. S4a, b). Next, we examined STAT3 activation in an HBCx34 PDX model that has been selected for tamoxifen resistance, which we previously demonstrated to display enrichment of breast CSCs [12]. Increased phospho-STAT3 was seen compared with the parental endocrine-sensitive HBCx34 PDX model and this was reversed by treatment with SFX-01 (Fig. 4b). Pull-down experiments with an activity-based probe (ABP) of SFN [21] in HBCx34 PDX tumor cells confirmed direct interaction of SFN with STAT3 in both antiestrogen and vehicle-treated tumors (Fig. 4c), establishing STAT3 as the likely target of SFX-01.

Next we tested whether SFX-01 inhibited STAT3 signaling in antiestrogen resistant patient samples. Cells from metastatic sample BB3RC61 treated with SFX-01 showed a significant reduction in phospho-STAT3 expression compared with tamoxifen or fulvestrant treatments alone (Fig. 4d). Furthermore, we observed direct binding of the sulforaphane activity-based probe (SFN-ABP) to STAT3 in cells from this same patient sample (Fig. 4e). Analysis of four additional antiestrogen resistant metastatic samples confirmed that SFX-01 combined with either tamoxifen or fulvestrant inhibited STAT3 activity in three of these patient samples (BB3RC44, BB3RC66, and BB7103), though SFX-01 only inhibited fulvestrant-induced STAT3 activation in BB3RC66 sample (Fig. S4c). Interestingly, SFX-01 did not repress STAT3 activity in sample BB7121 that had previously been treated clinically with SFX-01 (STEM trial). This patient’s disease had progressed in the presence of SFX-01 and was thus resistant to it.

On the whole, we have established, using PDX models in vivo and patient samples in vitro, that SFX-01 targets STAT3 and can inhibit its activity that is induced by treatment with antiestrogens.

### STAT3-related genes are up-regulated in antiestrogen resistant ALDH+ cells and are associated with worse outcomes for ER+ breast cancer patients

**Fig. 5:**
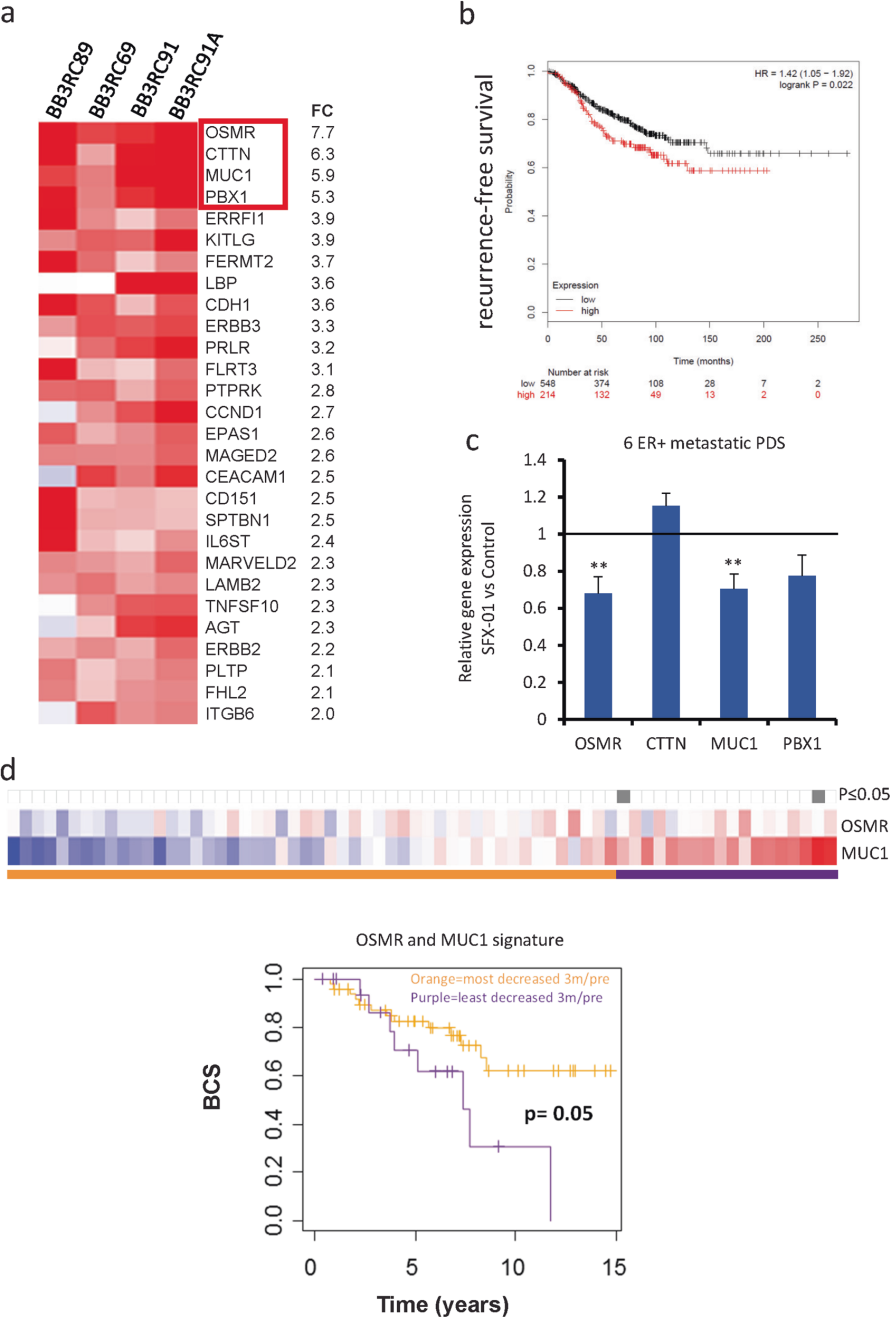
STAT3-related genes are up-regulated in antiestrogen resistant ALDH+ cells and are associated with worse outcomes for ER+ breast cancer patients. **a** Heatmap illustrating the 28 STAT3-related genes differentially expressed in ALDH+ cells of four ER+ metastatic patient-derived cells treated with antiestrogen treatments (fold change ≥2, pairwise Rank Products). Red color represents gene up-regulation while blue shows down-regulation in ALDH+ relative to ALDH- cells. **b** Kaplan–Meier analysis of high versus low expression of STAT3 28-gene signature showing recurrence-free survival in ER+ breast cancer patients. The gene expression data is from published microarray datasets available in KM plotter [25]. **c** Expression of *OSMR, CTTN, MUC1,* and *PBX1* genes was assessed by real-time qPCR analysis and normalized to control to calculate fold change. Metastatic patient-derived cells were treated for 72 h with SFX-01 (5 μM) or water (control). **d** Heatmap showing *OSMR* and *MUC1* gene expression in patient matched dataset after 3 months treatment with letrozole relative to baseline. Heatmap is ranked from left to right using the sum of the expression of the two genes. Red indicates up-regulation and blue indicates down-regulation in treated tumors relative to baseline. All significant cut-points (*p* ≤ 0.05) are shown in gray. The Kaplan–Meier plot demonstrates that elevated expression of these genes is significantly associated with decreased breast cancer specific survival (BCS). Vertical bars on survival curves indicate censored cases. *p* value is based on a log-rank (Mantel–Cox) test.

To investigate which genes are regulated by STAT3 in the antiestrogen resistant ALDH+ CSC population, we compared the gene expression profile between ALDH+ and ALDH− cells of four metastatic ER+ patient samples treated with endocrine therapy prior to analysis (Fig. S5a) [24]. Ingenuity Pathway Analysis (IPA) demonstrated differential expression (fold change ≥2) of 28 STAT3-related genes that were all up-regulated in ALDH+ cells (Fig. 5a). Next, we hypothesised that this 28-gene STAT3 signature of ALDH+ cells could predict prognosis of patients diagnosed with ER+ BC. In published gene expression microarray datasets from 762 ER+ tumors (KM plotter, [25]), we found that elevated expression of the 28 STAT3-related genes before treatment was significantly associated with BC recurrence (Fig. 5b). Thus, activation of STAT3 signaling in ER+ tumors is associated with more aggressive disease.

We therefore tested whether SFX-01 could reduce expression of the top four STAT3 related genes in ER+ antiestrogen resistant patient samples, and we found that two of the genes (*OSMR* and *MUC1*) were significantly down-regulated by SFX-01 treatment (Fig. 5c). Based on these findings, we investigated if failure to reduce expression of these two STAT3 genes with antiestrogen treatments was associated with worse outcomes due to STAT3 activation in a 68-patient matched dataset [26] before and after 3 months treatment with an aromatase inhibitor. Remarkably, increase in the sum of these two genes (*OSMR* and *MUC1*) upon antiestrogen treatment was significantly associated with decreased BC specific survival (Fig. 5d). The change in expression of each of these genes individually was not significantly associated with outcome (data not shown).

Thus, persistent activation of the STAT3 signaling pathway after antiestrogen treatment could potentially be used as a biomarker to predict endocrine resistance and, hence, to identify the patient group most likely to benefit from STAT3 inhibition with SFX-01 therapy.

## Discussion

In this study, we establish that SFX-01 targets antiestrogen resistant breast CSCs in patient samples and PDXs. Compared with antiestrogen treatment alone, SFX-01 combined with antiestrogen drugs significantly reduced ALDH+ CSCs, their sphere-forming activity and tumor-initiating cell frequency, as well as formation of lung micrometastases in PDX tumors grown in mice. Mechanistically, both tamoxifen and fulvestrant induced STAT3 phosphorylation although not always to the same degree in individual tumors. We show that SFX-01 suppresses this adaptive STAT3 phosphorylation and establish that an ABP of SFN directly binds to and targets STAT3 in cells from patient samples and PDX tumors. Importantly, analysis of ALDH+ cells from endocrine-resistant patient samples revealed activation of STAT3 signaling and expression of STAT3 target genes *MUC1* and *OSMR*, which were down-regulated by SFX-01 in patient samples. Moreover, increased expression of these two genes after 3 months’ endocrine treatment predicted poor prognosis in patients with ER+ tumors. We propose that inhibiting STAT3 signaling with SFX-01 may help to overcome endocrine therapy resistance and recurrence in ER+ BC.

We previously reported that CSC activity is increased by the antiestrogens tamoxifen and fulvestrant, both in vitro and in vivo [12], and others have demonstrated a role for SFN in targeting breast CSCs in vitro with concentrations ranging 0.5–5 μM [14]. Here, we show that a stabilised form of SFN, SFX-01, is a potent inhibitor of endocrine resistant CSCs using an equivalent dose of 5 μM. In contradistinction to the previous report that used cell lines [14], we establish that STAT3, not Wnt signaling, is the primary target mechanistically responsible for endocrine resistance of breast CSCs in human breast tumors.

STAT3 activation has been associated with ALDH expression and MFE in triple negative BC cell lines [23, 27] and recently it has been shown that a STAT3 inhibitor blocking phosphorylation can target breast CSCs in ER+ palbociclib-resistant cell lines [28]. We previously reported that Notch4 signaling is increased in antiestrogen resistant ALDH+ breast CSCs [12], and using a tamoxifen-resistant MCF7 sub-line, an association between Notch4 and STAT3 activation was established [22]. However, we have not confirmed here that STAT3 phosphorylation is confined to the ALDH+ population. The relevance of our finding is strengthened by demonstrating that STAT3 is activated in both antiestrogen treated PDX tumors and patient-derived samples.

The recent finding that SFN bound directly to STAT3 with high-affinity in BC cell lines [21] led us to investigate STAT3 as the target in antiestrogen treated PDX and endocrine resistant patient-derived samples. We show convincingly that binding occurs and, since SFN is known to react with cysteine residues [29], future research is warranted to determine precisely which STAT3 cysteine residues are targeted by SFN. We also show that SFX-01 reduces the phosphorylation of STAT3 in vitro and in vivo, in short-term antiestrogen treatment and established resistance. This demonstrates its utility and provided a rationale for its use in clinical trials in patients, where plasma levels of about 1 µM SFN are achieved following SFX-01 administration (data not shown). Therefore, we recently recruited 47 patients with metastatic ER+ BC who were progressing on endocrine therapy and showed that the addition of SFX-01 to the endocrine therapy resulted in clinical benefit in 25% of patients [30]. Our in vivo data demonstrate where primary tumor phospho-STAT3 is reduced, there is a reduction in metastatic lung colonisation. This indicates that SFX-01 could reduce distant recurrence rates in early BC.

In order to better target STAT3-driven endocrine resistant BC, we analysed gene expression in samples from advanced BC patients who had received prior endocrine therapy. We derived a STAT3 signature consisting of 28 genes that was associated with poor progression-free survival of ER+ BC patients. Importantly, two of the most expressed genes, *OSMR* and *MUC1*, were down-regulated by SFX-01 treatment of patient-derived samples. Our results support these effects on *OSMR* or *MUC1* being most likely mediated by SFX-01 inhibition of STAT3 but we cannot rule out possible direct effects of SFX-01 on these two genes. Interestingly, *OSMR* STAT3-mediated transcription has recently been shown to be regulated by miRNA 551b-3p in BC cells [31], while STAT3 transcriptional activation of *MUC1* occurs through a direct association with a STAT3-binding site in the *MUC1* promoter [32]. *OSMR* and *MUC1* were both STAT3-responsive and SFX-01-sensitive in patient samples, and their increased expression after 3 months’ endocrine treatment accurately predicted survival over a 14 year period after surgery for BC. These data suggest that this two-gene signature could have potential to select tumors responsive to SFX-01, which would have to be confirmed in a larger group of patients.

*OSMR* has already been associated with worst prognosis although not specifically for ER+ tumors [33], and an association between OSMR signaling and reduced ER levels has been reported [33]. Moreover, in silico data of breast tumors shows a significant relationship between OSMR and markers of CSCs [34] and OSMR signaling increases invasive capacity of BC cell lines [35]. In addition, MUC1 has been shown to contribute to the self-renewal of BC cells inducing MFE [36], ALDH activity [37], and tamoxifen resistance [38], as well as predicting failure to tamoxifen treatment [39].

Thus, overall we establish the potential of SFX-01 for clinically meaningful improvements to endocrine therapy in ER+ BC by inhibiting STAT3 signaling and reversing CSC-mediated resistance. The derivation of a STAT3 pathway signature that predicts poor prognosis and resistance to endocrine therapy indicates that a subpopulation of ER+ breast tumors might be the rational targets for SFX-01 therapy in combination with current endocrine therapies.

## Materials and methods

### Breast cancer patient-derived samples

Early BC samples were collected from patients undergoing surgical resection of the primary tumor at three NHS Foundation Trusts (South Manchester, Salford Royal, and The Pennine Acute Hospitals). Metastatic fluids (ascites or pleural effusions) drained from advanced BC patients were collected at the Christie and South Manchester Hospitals NHS Foundation Trusts through the Manchester Cancer Research Centre Biobank. Fully informed consent from all patients was obtained in accordance with local National Research Ethics Service guidelines (study numbers: 05/Q1402/25, 05/Q1403/159, and 12/ROCL/01). Early and metastatic BC samples were processed as described previously [12, 20]. Supplementary Tables 1 (early BC) and two (metastatic BC) present the clinico-pathological characteristics of the samples used in this study.

### In vivo experiments using patient-derived xenografts

All in vivo studies were carried out in accordance with the UK Home Office (Scientific Procedures) Act 1986 under project licence PPL40/3645. NOD *scid gamma* (NOD.*Cg-Prkdc*^scid^ Il2rg^tm1Wjl^/SzJ, NSG) and athymic Nude (Foxn1^nu^) mice (Charles River) were used in the following experiments. Small fragments of HBCx34 (early) and BB3RC31 (metastatic) BC PDX tumors were implanted subcutaneously into the flanks of 8–12-week-old female NSG mice. These two preclinical models are estrogen-dependent, so animals were administered with 8 μg/ml 17-beta estradiol (Sigma-Aldrich, #E2758) in drinking water at least 4 days previously to implantation until the end of the experiment. HBCx34 PDX model and their endocrine resistant variant (tamoxifen-resistant, TAMR) were kindly provided by Dr Elisabetta Marangoni (Institut Curie, Paris) [19]. In vivo experiments with HBCx34 TAMR PDX model were carried out using Nude mice in the Institut Curie. For clinical details of BB3RC31 refer to [12, 20].

When average PDX tumor volume reached 200–300 mm^3^, mice were randomized between treatment groups ensuring that all groups had similar starting mean tumor volumes. All in vivo experiments were performed with a minimum of *n* = 3 mice per condition. Tumor size and animal weight were recorded twice weekly in a blinded manner. SFX-01 (Evgen Pharma PLC, 300 mg/kg/day) and tamoxifen citrate (Sigma-Aldrich, #T9262, 10 mg/kg/day) were administered by oral gavage (0.1 ml/dose) on a 5 day from 7 day basis (weekends excluded) for 14 or 56 days; whereas Fulvestrant (200 mg/kg/week, Astrazeneca) was injected subcutaneously (0.1 ml/dose) on a weekly basis for 56 days. SFX-01 and tamoxifen citrate were made in 1% carboxymethylcellulose (Sigma-Aldrich, #C9481) dissolved in distilled water. SFN levels were measured in the mice plasma achieving a concentration of about 2 µM (data not shown), which is an appropriate approximation of the in vitro dose used (5 µM) since SFN is rapidly metabolized in vivo [40]. Upon termination, PDX tumors were collected in ice-cold DMEM media and processed as described in [12] for further downstream analyses. Mouse lungs were formalin-fixed paraffin-embedded for histological assessment of metastatic disease.

In vivo limiting dilution assays were performed to evaluate tumor initiation ability of HBCx34 PDX after 2 weeks of in vivo treatment. Xenografts treated with either SFX-01, tamoxifen citrate, combination or vehicle control for 14 days were collected and digested using collagenase-hyaluronidase (Stem Cell Technologies) to obtain single-cell suspensions. Serial limiting dilution of PDX-derived cells (500,000; 100,000; 20,000; 4,000 cells) were resuspended in mammosphere media:Matrigel (1:1) and subcutaneously injected into the flank of NSG mice (*n* = 4 per condition). The 90-day slow-release estrogen pellets were implanted subcutaneously 2 days prior to cell injection (0.72 mg, Innovative Research of America). At day 90 after cell injection, positive tumor growth was considered in mice bearing a tumor greater than 75 mm^3^. The tumor-initiating cell frequency was calculated using Extreme Limiting Dilution Analysis software (The Walter and Eliza Hall Institute of Medical Research) with a 95% confidence interval. *p* values were obtained by Chi-squared statistical analysis.

### Mammosphere colony assay

Cancer stem cell activity using the mammosphere colony assay was determined following the protocol described elsewhere [41]. Freshly isolated cells from ER+ primary and metastatic patient-derived samples (500 cells/cm^2^) were cultured for 7–9 days in mammosphere culture conditions. When indicated, cells were either treated directly in the mammosphere media or pre-treated for 72 h in low adherence previously to assess their mammosphere forming ability. PDX-derived cells (500 cells/cm^2^) were cultured for 7–10 days. Cell lines were pre-treated in adherence for 3 days and then cultured in suspension for 5 days (200 cells/cm^2^). Mammosphere formation efficiency (MFE, %) was calculated by dividing the number of mammospheres formed per well (≥50 μm diameter) by the number of single cells seeded per well. MFE is expressed as the average percentage of MFE.

### ALDEFLUOR assay

Cancer stem cell activity was measured using the ALDEFLUOR assay (Stem Cell Technologies). This fluorescent reagent system allows detecting the activity of aldehyde dehydrogenase (ALDH), enzyme highly active in mammary stem cells [17]. The assay was performed following the manufacturer’s instructions. Single cells were resuspended in ALDEFLUOR assay buffer containing 1.5 mM bodipyaminoacetaldehyde, ALDH substrate, and incubated in the dark for 45 min at 37 °C. In parallel, a fraction of the cells was incubated in the presence of a twofold molar excess of the ALDH inhibitor, diethylaminobenzaldehyde (DEAB), in order to correct for background staining and define the ALDH-positive region during the analysis. When using PDX tumors, a Pacific Blue anti-mouse H-2K^d^ MHC Class I antibody (BioLegend, #116616) was used to remove mouse cells from the analysis. In all cases, dead cells were excluded using 7-aminoactinomycin D (7AAD, BD Biosciences). Data were acquired with the BD LSRII flow cytometer (BD Biosciences) and analysed using the BD FACSDiva^TM^ software.

### Western blot

Cells were lysed and samples prepared for Western blot as explained in [12], detailed steps are shown in Supplementary Materials and Methods. Primary antibodies used were: Phospho-STAT3 Y705 (Cell Signaling, #D3A7), STAT3 (Cell Signaling, #124H6), phospho-NFκB p65 S536 (Cell Signaling, #93H1), NFκB (Cell Signaling, #D14E12), β-actin (Sigma-Aldrich, #A2228).

### Affinity pull-down assays

SFN-ABP was prepared and used in affinity pull-down assays following the approach described in [21]. Further details can be found in Supplementary Materials and Methods.

### Immunohistochemistry

Formalin-fixed paraffin-embedded PDX tumors and mouse lung tissue were cut into 4 μm-thick sections for immunohistochemical analysis. Sections were stained for either human Ki67 (Dako, #M7240) [12] or anti-human mitochondrial antibody (Abcam, #ab92824) [20]. Further details are included in Supplementary Materials and Methods.

### Gene expression analysis of ALDH+/− populations in ER+ metastatic breast cancer

Whole transcriptomic datasets for ALDH+ and ALDH− populations from ER+ metastatic patient-derived samples were available here [24]. Briefly, ER+ advanced BC patients were drained of metastatic fluids (ascites or pleural effusions) due to discomfort. Breast cancer cells were isolated following the methodology described elsewhere [20] and then stained using the ALDEFLUOR assay. After FACS sorting, RNA was extracted and the whole transcriptome of ALDH+ and ALDH- populations was assessed using Affymetrix Whole-Transcript Human Gene 1.0 ST Array (Affymetrix, Thermo Fisher Scientific) [24]. Four patient samples were selected on the basis of being ER+ PR+ HER2− and having received endocrine treatment prior to collection. Differentially expressed STAT3-related genes (upstream and downstream) between ALDH+ and ALDH− cells were identified using IPA (Qiagen).

### Quantitative real-time PCR

Six metastatic patient-derived samples were treated in adherence for 72 h in the presence of 5 μM SFX-01 or water (vehicle control) in DMEM/F-12 GlutaMAX medium (GIBCO) containing 10% fetal bovine serum (FBS; GIBCO), 10 mg/ml insulin (Sigma-Aldrich), 10 mg/ml hydrocortisone (Sigma-Aldrich), and 5 ng/ml epidermal growth factor (EGF; Sigma-Aldrich). Total RNA was then extracted as described in [12]. RNA quality, integrity, and concentration were assessed using the Qubit RNA HS assay and the Agilent Bioanalyzer. Gene expression was evaluated with the 48.48 IFC Dynamic Arrays (Fluidigm Corporation) using standard Taqman Assays as per protocol (see Supplementary Materials and Methods for further information).

### Statistical analysis

Unless otherwise stated, statistical analyses were carried out by a two-tailed Student’s *t* test. A *p* value ≤ 0.05 was considered to be statistically significant. Error bars represent the standard error of mean (SEM) of at least three independent experiments. Data are shown as mean ± SEM.

Survival analysis was performed on patient datasets using either the KM plotter online tool [25] or performing Cox proportional hazards tests for all possible points-of-separation using the survivALL R package [42].

## Supplementary information

Supplementary Figure S1

Supplementary Figure S2

Supplementary Figure S3

Supplementary Figure S4

Supplementary Figure S5

Supplementary Table 1

Supplementary Table 2

Supplementary Figure Legends

Supplementary Materials and Methods
