# Supplementary figures and images for: Targeting STAT3 signaling using stabilised sulforaphane (SFX-01) inhibits endocrine resistant stem-like cells in ER-positive breast cancer
Source: Oncogene. 2020 May 30;39(25):4896–908. doi: 10.1038/s41388-020-1335-z (PMC7299846; doi:10.1038/s41388-020-1335-z)

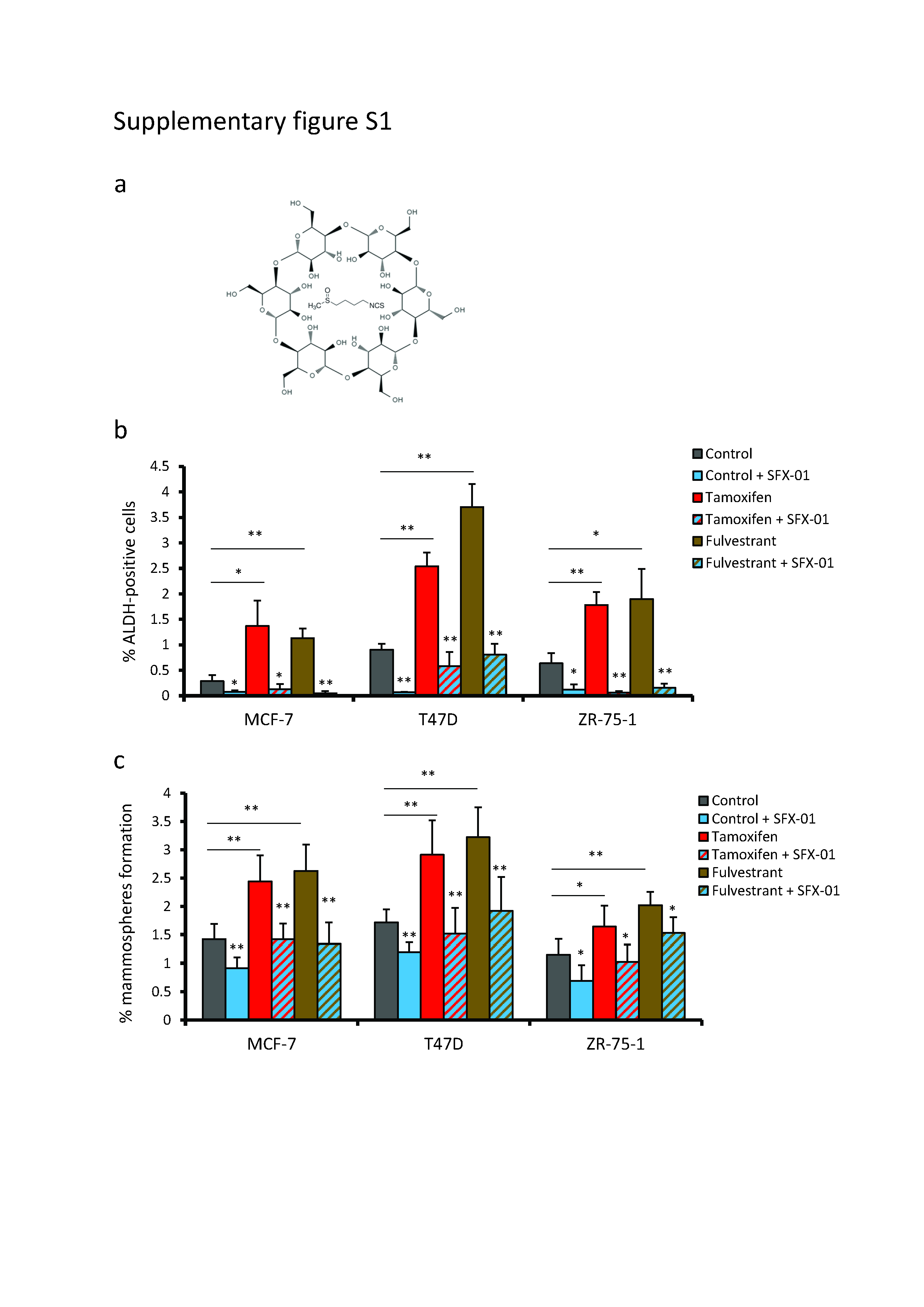

Supplement: Supplementary file 1 — Supplementary Figure S1 [file 41388_2020_1335_MOESM1_ESM.tif]

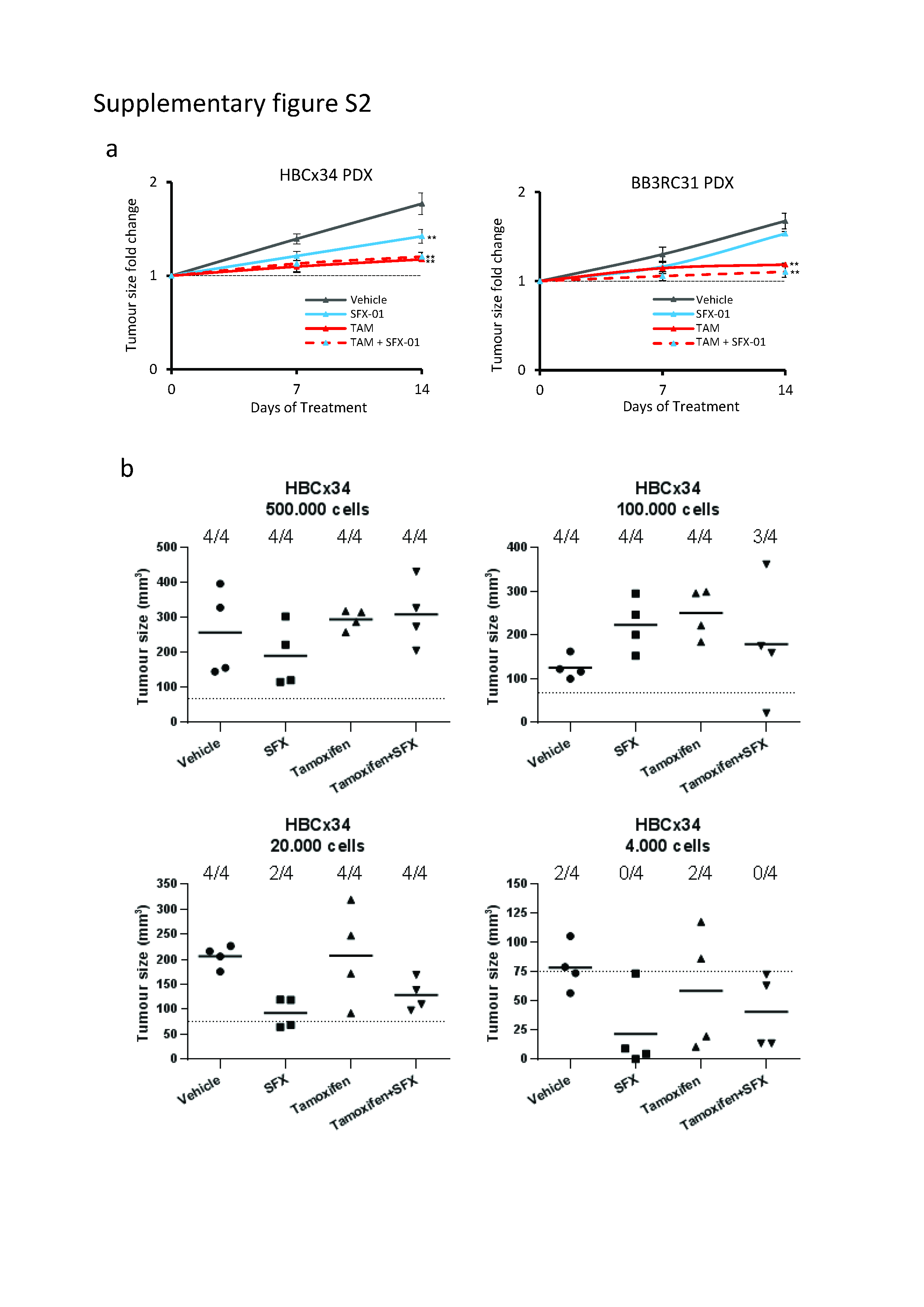

Supplement: Supplementary file 2 — Supplementary Figure S2 [file 41388_2020_1335_MOESM2_ESM.tif]

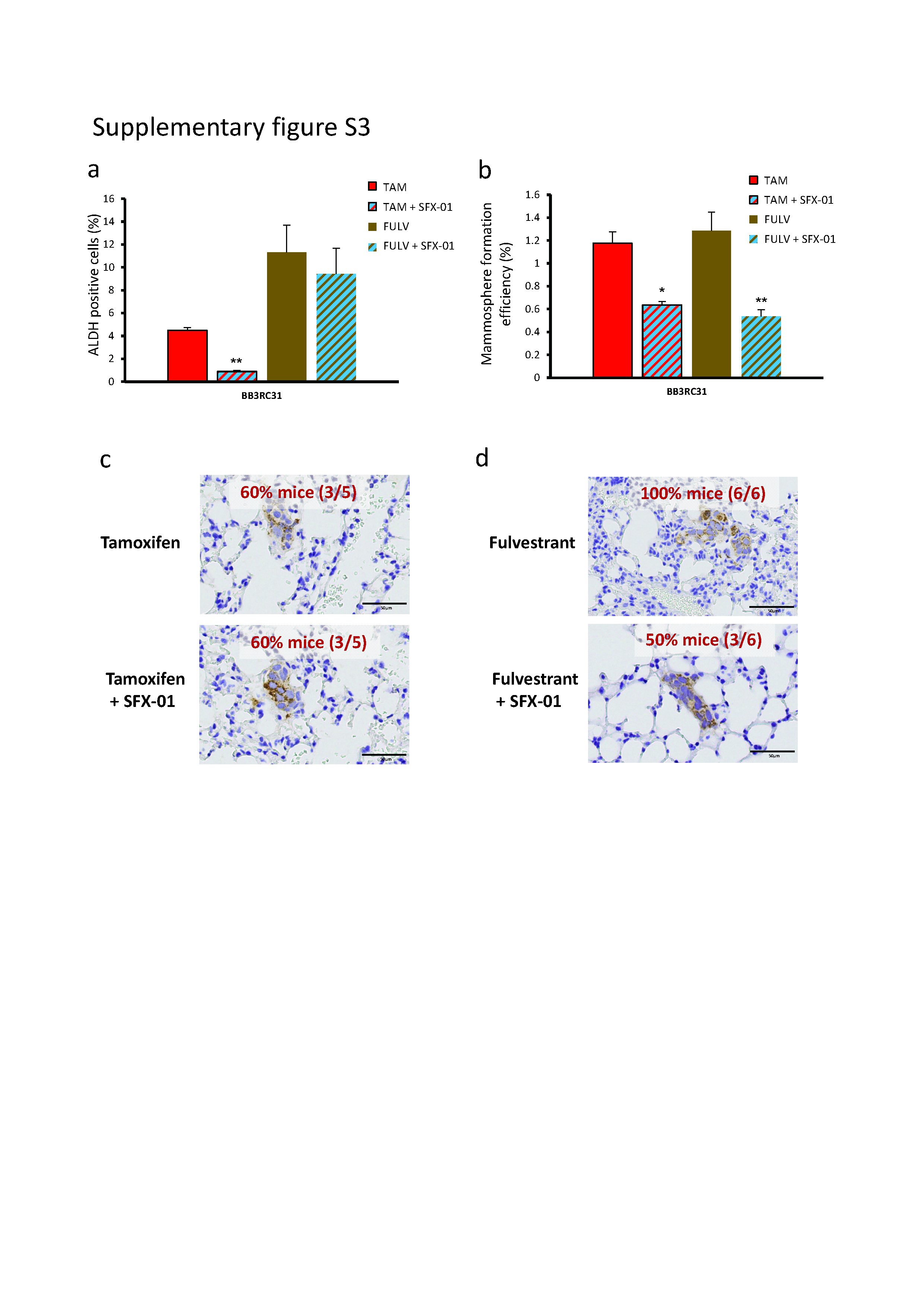

Supplement: Supplementary file 3 — Supplementary Figure S3 [file 41388_2020_1335_MOESM3_ESM.tif]

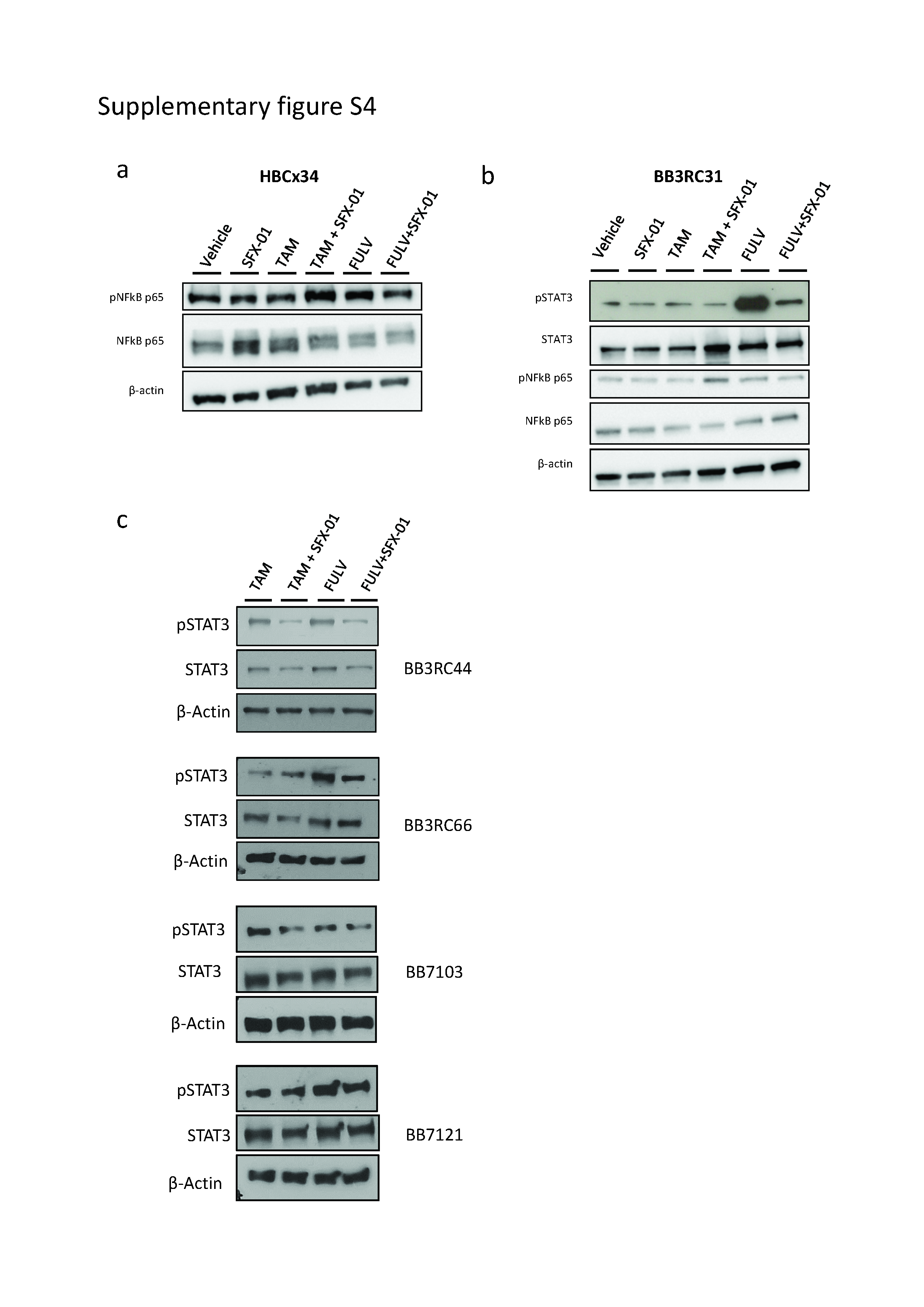

Supplement: Supplementary file 4 — Supplementary Figure S4 [file 41388_2020_1335_MOESM4_ESM.tif]

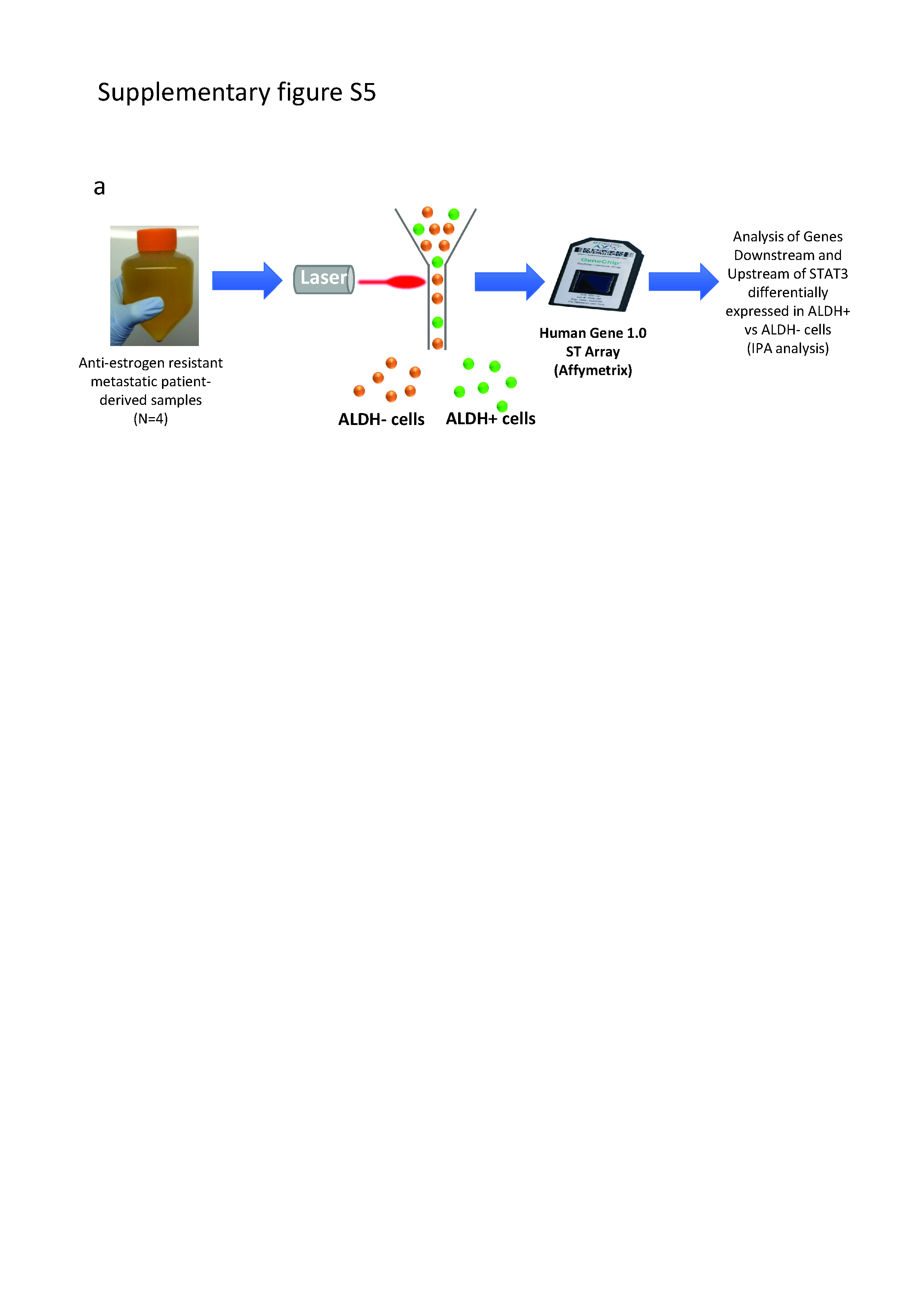

Supplement: Supplementary file 5 — Supplementary Figure S5 [file 41388_2020_1335_MOESM5_ESM.tif]

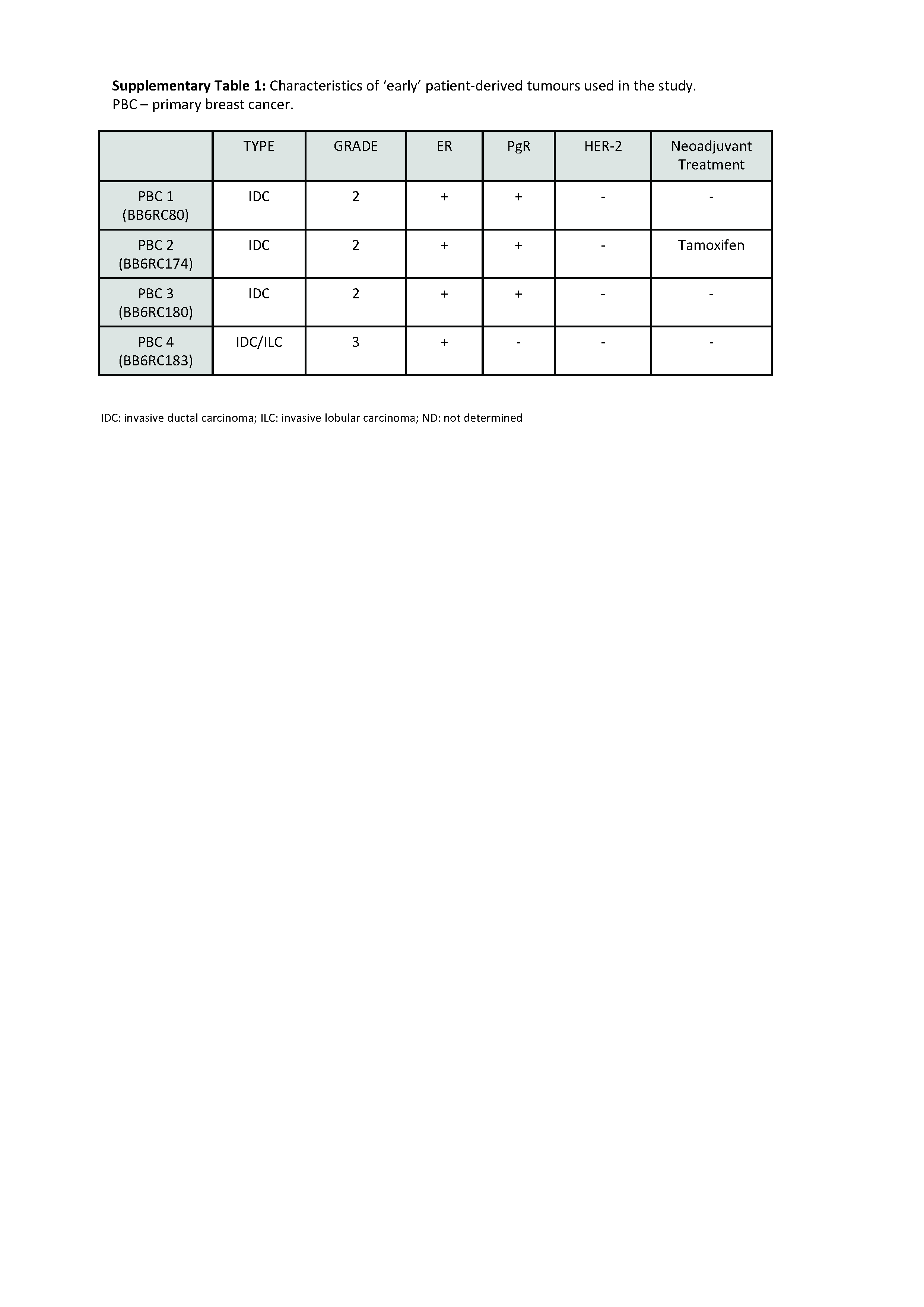

Supplement: Supplementary file 6 — Supplementary Table 1 [file 41388_2020_1335_MOESM6_ESM.tif]

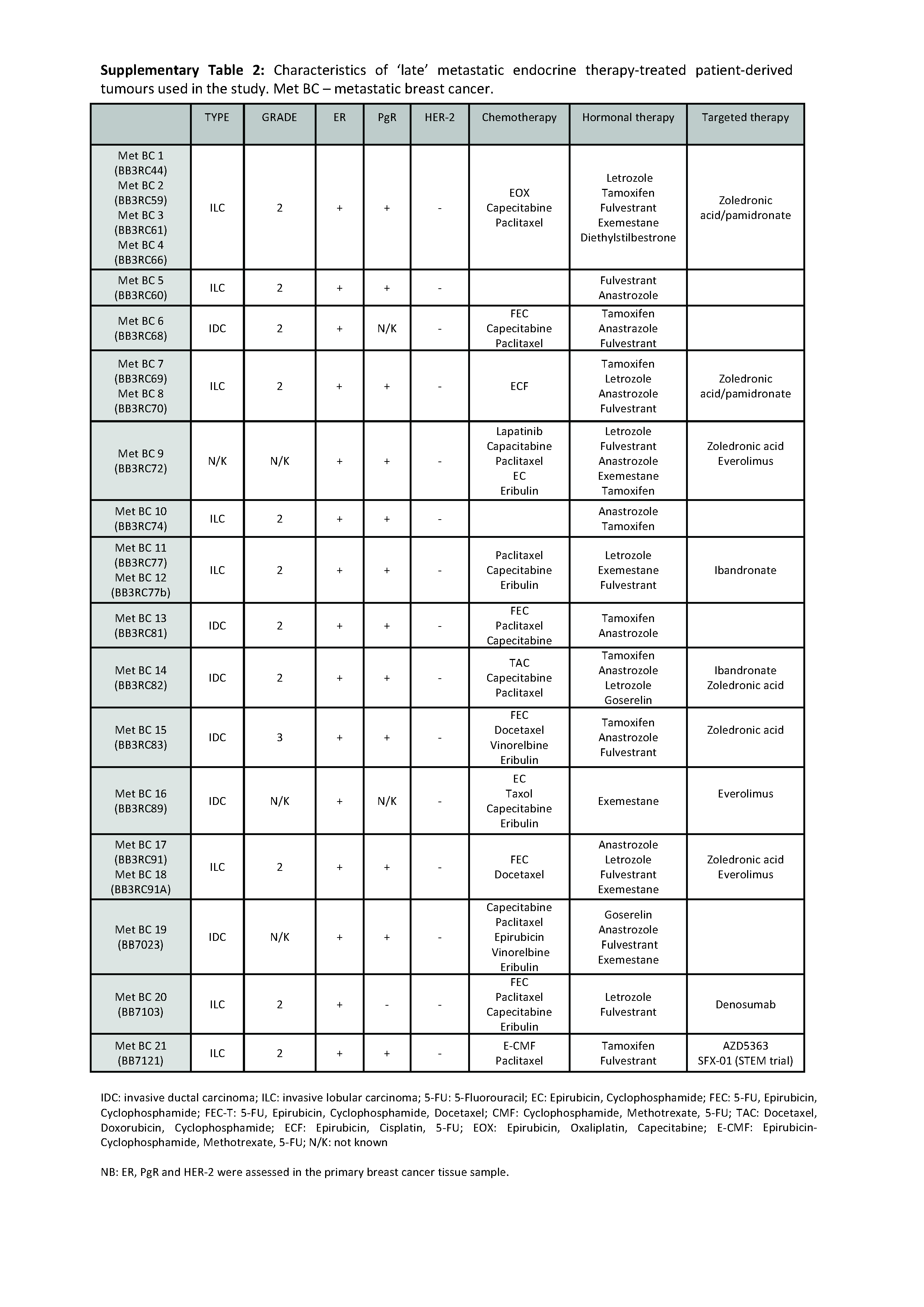

Supplement: Supplementary file 7 — Supplementary Table 2 [file 41388_2020_1335_MOESM7_ESM.tif]
