# Supplementary material for: Targeting STAT3 signaling using stabilised sulforaphane (SFX-01) inhibits endocrine resistant stem-like cells in ER-positive breast cancer
Source: Oncogene. 2020 May 30;39(25):4896–908. doi: 10.1038/s41388-020-1335-z (PMC7299846; doi:10.1038/s41388-020-1335-z)
Supplement: Supplementary file 8 — Supplementary Figure Legends [file 41388_2020_1335_MOESM8_ESM.docx]

**SUPPLEMENTARY FIGURE LEGENDS**

**Figure S1**

1. Graphic representation of chemical structure of SFX-01 showing synthetic sulphoraphane molecule stabilised in an alpha-cyclodextrin complex.
2. Mammosphere formation efficiency of MCF-7, T47D and ZR75-1 cells. Cells were pre-treated in adherence with ethanol (Control), tamoxifen (1 μM) or Fulvestrant (0.1 μM) in the presence of SFX-01 (5 μM) or water for 72 hours, and then plated at low density (200 cells/cm^2^) into 6-well plates and allowed to grow in suspension for 5 days. Mammosphere formation efficiency was calculated by dividing the number of mammospheres formed (≥ 50μm diameter) by the original number of single cells seeded and is expressed as the mean percentage of mammosphere formation ± standard deviation.
3. FACS analysis of ALDH1 enzymatic activity assessed by the ALDEFLUOR assay in MCF-7, T47D and ZR75-1 cells. Graphs show the mean percentage of ALDH-positive cells (± standard deviation) for each cell line grown and treated as in A). After 72 hours of treatment, ALDEFLUOR assay was performed.

p values refer to SFX-01 treatment compared to respective non-SFX-01 bars, * p < 0.05, ** p < 0.01

**Figure S2**

1. Early (HBCx34) and metastatic (BB3RC31) PDXs tumor size variation over 14 days *in vivo* treatments with SFX-01 (300mg/kg/day, oral gavage) in the presence or absence of tamoxifen (10mg/kg/day, oral gavage). Tumor size was determined every 3-4 days and averaged for each week. Fold change was calculated by dividing the tumor size by the size of the respective tumor at day 0.
2. Graphs representing tumor size at day 90 after cell injection for each cell number tested. HBCx34 cells were pre-treated *in vivo* for 14 days. Experiments (N=4 per condition) were carried out in NSG mice injected subcutaneously with 500 000, 100 000, 20 000 and 4 000 cells. 90-day slow release estrogen pellets were implanted subcutaneously into mice two days before cell injection (0.72 mg, Innovative Research of America). Positive tumor growth was assessed by determining the mice bearing a tumor greater than 75 mm^3^ and is represented as mice positive for growth/mice tested.

** p < 0.01

**Figure S3**

BB3RC31 PDX treated *in vivo* for 56 days with tamoxifen (10mg/kg/day, oral gavage) or fulvestrant (200mg/kg/week, subcutaneous injection) in the presence or absence of SFX-01 (300mg/kg/day, oral gavage).

1. Percentage of ALDH-positive cells was determined with ALDEFLUOR assay. ALDH-positive cells were discriminated from ALDH-negative cells using the ALDH inhibitor, DEAB.
2. Mammosphere formation efficiency was determined on day 7-9 and calculated by dividing the number of mammospheres formed (≥ 50μm diameter) by the original number of single cells seeded (500 cells/cm^2^) and is expressed as the mean percentage of mammosphere formation.

**c-d** Mice lungs were stained with anti-human mitochondrial antibody and micrometastases with at least 10 cells were counted. Percentage of mice bearing micrometastases for each tamoxifen (C) and fulvestrant (D) treatment group is shown.

Data is represented as mean ± SEM. * p < 0.05; ** p < 0.01

**Figure S4**

**a**  phospho-NFkB p65 and total NFkB p65 protein expression levels determined by Western Blot in HBCx34 PDX treated *in vivo* for 56 days with tamoxifen or fulvestrant in the presence or absence of SFX-01. β-actin was used as a reference for the loading control.

**b** phospho-STAT3, total STAT3, phospho-NFkB p65 and total NFkB p65 protein expression levels determined by Western Blot in BB3RC31 PDX treated *in vivo* for 56 days with tamoxifen or fulvestrant in the presence or absence of SFX-01.

**c**  phospho-STAT3 and total STAT3 protein expression levels in four metastatic patient-derived samples treated for 72 hours with tamoxifen or fulvestrant in the presence or absence of SFX-01.

**Figure S5**

1. Schematic overview of the experimental approach used to profile gene expression of ALDH+ and ALDH- cells from metastatic patient samples. Ingenuity Pathway Analysis (IPA) software was employed to determine STAT3-related genes differentially expressed in ALDH+ cells.
