# Supplementary material for: Targeting STAT3 signaling using stabilised sulforaphane (SFX-01) inhibits endocrine resistant stem-like cells in ER-positive breast cancer
Source: Oncogene. 2020 May 30;39(25):4896–908. doi: 10.1038/s41388-020-1335-z (PMC7299846; doi:10.1038/s41388-020-1335-z)
Supplement: Supplementary file 9 — Supplementary Materials and Methods [file 41388_2020_1335_MOESM9_ESM.docx]

**SUPPLEMENTARY MATERIALS AND METHODS**

Cell lines and reagents

MCF7, T47D and ZR-75-1 cell lines were obtained through the American Tissue Culture Collection (ATCC) and cultured in Dulbecco’s Modified Eagle’s Medium/Nutrient Mixture F-12 Ham (DMEM/F12) with GlutaMAX (Gibco) containing 10% foetal bovine serum (Gibco). Cell lines are routinely authenticated (PowerPlex 21 Kit for STR profiling, Promega) and monthly screened for mycoplasma (Venor®GeM-qEP Mycoplasma Detection Kit, CamBio) at Molecular Biology Core Facility at the Cancer Research UK-Manchester Institute. All tests performed were negative for mycoplasma contamination. Cells were pre-treated in adherence for 3 days with either ethanol (control), 1μM 4-OH-tamoxifen (Sigma-Aldrich) or 0.1μM fulvestrant (TOCRIS Bioscience) in the presence of 5μM SFX-01 (Evgen Pharma) or water (control), respectively. The molar concentration of SFX-01 is the same as SFN since there is one molecule of SFN per molecule of alpha-cyclodextrin.

Western blot

Cells were resuspended in Protein Lysis Buffer (25 mM HEPES, 50 mM NaCl, 50 mM sodium pyrophosphate, 50 mM sodium fluoride, 1% Triton-X-100, 10% glycerol, 5 mM EDTA, Roche MiniProtease Inhibitor cocktail tablet, 1 μM PMSF) and placed on a rotator for 1 hour at 4°C. Protein lysates were then obtained by centrifugation at 10,000×g for 10 min at 4°C. Protein concentration was determined using the BCA Protein Assay kit (Thermo Fisher Scientific). Samples were loaded in pre-cast 10% gels for SDS-PAGE (BioRad, #456-1033) and run at 200 V for 1 hour. Proteins were then wet-transferred to a 0.2 μm Nitrocellulose membrane (Amersham GE Healthcare) at 150 V for 1 hour. Membranes were blocked in 5% skimmed milk or bovine serum albumin (BSA)/TBS- 0.001 % Tween 20 (TBST-T) for 1 hour at room temperature followed by an overnight primary antibody incubation at 4°C. Following 3 washes with TBS-T, membranes were incubated with HRP-conjugated secondary antibodies (Dako #P0447, #P0448, #P0449) for 1 hour at room temperature. Blots were developed with Luminata Classico or Luminata Forte Western HRP Substrate (EMD Millipore, #WBLUC0100, # WBLUF0100) by exposing the membranes to hyperfilm (Amersham GE Healthcare).

Immunohistochemistry

Using the Bond Automated Stainer (Leica Biosystems), tumor sections were stained for human Ki67 (Dako, #M7240) using the Target Retrieval Solution pH 9.0 (Dako, #S2367). The antibody was detected using Dako EnVision Detection System Peroxidase/DAB (Dako, #K5007). Definiens Tissue Studio software was used to quantify the staining. The percentage of positive epithelial cells was scored at least on 3 different tissue samples.

PDX lungs were examined for metastatic lesions of human origin by staining with anti-human mitochondrial antibody (Abcam, #ab92824). At least 2× step sections per lung were stained on a Bond Automated Stainer (Leica Biosystems) using standard protocol F and Target Retrieval Solution pH 6.0 (Dako) for 20 min. Direct visual examination of stained sections was carried out by an unbiased observer to detect micrometastases. Lesions with at least 10 cells were classed as micrometastases.

All sections were counterstained with haematoxylin.

Quantitative Real-Time PCR using the Biomark HD System (Fluidigm)

Gene expression was evaluated with the 48.48 IFC Dynamic Arrays (Fluidigm Corporation) using standard Taqman Assays as per protocol (PN 68000089 H1). Taqman Gene Expression Assays used: *CTTN* (Hs01124232_m1), *MUC1* (Hs00159357_m1), *OSMR* (Hs00384276_m1), *PBX1* (Hs00231228_m1), *PGK1* (Hs99999906_m1), *SDHA* (Hs00188166_m1) (Thermo Fisher Scientific). cDNA was prepared with Reverse Transcription Master Mix (Fluidigm, #100-6297). Samples were incubated on a thermal cycler (MJ Research) for 5 min at 25°C, 30 min at 42°C and 5 min at 85°C. For the pre-amplification step (PN 100-5876 C2), a diluted pool of gene expression assays was prepared with the Dilution Reagent (10 mM Tris-HCl, pH 8.0, 1 mM EDTA, Fluidigm, #100-8726) and mixed with the Preamp Master Mix (Fluidigm, #100-5744) and cDNA. Samples were then incubated in a T100 thermal cycler (BioRad) for 2 min at 95°C followed by 14 cycles of 15 sec at 95°C and 4 min at 60°C. After cycling, resulting samples were diluted 1:5 with Dilution Reagent. Then each 20X Taqman Gene Expression Assay was diluted in 2X Assay Loading Reagent (Fluidigm, #100-7611). Sample pre-mix was made by combining Taqman Universal PCR Master Mix 2X (Life Technologies, #4304437), 20X GE Sample Loading Reagent (Fluidigm, #100-7610) and pre-amplified cDNA. Following hydraulic chip priming, the diluted assays and samples were transferred into the appropriate IFC inlets and loaded with the IFC MX Controller (Fluidigm). Then the loaded chip was placed in the Biomark HD instrument (Fluidigm) and run the GE 48x48 specific protocol, 96.5 °C for 10 min followed by 40 cycles at 96 °C for 15 sec and 60 °C for 1 min. The data were normalised to average of housekeeping genes and ΔCt, ΔΔCt and fold change values were calculated for each metastatic sample.

Affinity pull-down assays

Cells from patient-derived samples and xenografts were resuspended in serum-free DMEM media containing 5 μM SFN ABP probe and incubated for 30 min at 37°C. Following in-cell labelling with the probe, cells were washed with serum-free DMEM media and lysed in PBS pH 7.4 containing 1% Triton-X-100, 0.1% SDS, 1x Complete EDTA-free protease inhibitor tablet. After 5 min on ice, samples were sonicated and lysates were obtained upon centrifugation at 10,000 x g for 5 min at 4°C. Protein concentration was determined in the lysates and aliquots at 1 mg/ml were made with lysis buffer (100 μl per aliquot). Probe-labelled proteins were ligated to Copper-catalysed azide-alkyne cycloaddition (CuAAC), which was subsequently captured with azido-TAMRA-biotin (AzTB) (click reaction). Samples were vortexed for 1 hour at room temperature and then the click reaction was quenched by addition of 10 mM EDTA. Proteins were precipitated by centrifugation at 6,000×g for 4 min in methanol:chloroform:water (4:1:2). Protein pellets were then washed twice with 4 volumes methanol by vortexing, sonication and centrifugation at 8,000×g for 4 min. Protein pellets were air dried for 10 min before being resuspended in 0.2% SDS/PBS (1mg/ml, 100 μl), vortexed and sonicated until fully dissolved. 10 μl aliquot was taken at this stage as input. Then probe-labelled proteins were pulled-down using avidin-coated magnetic beads. After equilibrating the beads in 0.2% SDS/PBS, 10 μl of bead solution was added to samples, vortexed and incubated for 2-3 hours at room temperature. After brief centrifugation, samples were placed on magnet for 1 min and supernatant was removed carefully. 10 μl aliquot was taken at this stage as supernatant. Following several washes in 0.2% SDS/PBS, beads were boiled at 100°C for 5 min in the presence of Laemmli loading buffer containing β-mercaptoethanol and placed on magnet for 1 min. Probe-enriched samples were then transferred to a new tube and resolved by 12% gel SDS-PAGE. To check whether click reaction worked, TAMRA in-gel fluorescence was visualised using a Typhoon Biomolecular Imager (λ_exc_= 552 nm, λ_em_=570 nm; Amersham GE Healthcare). Thus, proteins were transferred to PDVF membranes using an iBlot Gel Transfer (Life Technologies) for 1 hour at 100 V. Then membranes were blotted and developed as mentioned above (see *Western blot* section) using as primary antibodies anti-STAT3 (Cell Signalling, #124H6) and anti-GAPDH (Abcam, #Ab9485).
